# HPV and Recurrent Respiratory Papillomatosis: A Brief Review

**DOI:** 10.3390/life11111279

**Published:** 2021-11-22

**Authors:** Amr Mohamed Ouda, Ahmed Adel Elsabagh, Ibrahim Mohamed Elmakaty, Ishita Gupta, Semir Vranic, Hamda Al-Thawadi, Ala-Eddin Al Moustafa

**Affiliations:** 1 College of Medicine, QU Health, Qatar University, Doha 2713, Qatar; ao1706425@qu.edu.qa (A.M.O.); ae1802661@qu.edu.qa (A.A.E.); ie1703006@qu.edu.qa (I.M.E.); ishita.gupta@qu.edu.qa (I.G.); svranic@qu.edu.qa (S.V.); halthawadi@qu.edu.qa (H.A.-T.); 2 Biomedical and Pharmaceutical Research Unit, QU Health, Qatar University, Doha 2713, Qatar; 3 Biomedical Research Centre, Qatar University, Doha 2713, Qatar

**Keywords:** recurrent respiratory papillomatosis, HPV, HPV infection, children, vaccine

## Abstract

Recurrent Respiratory Papillomatosis (RRP) is a rare but severe manifestation of human papillomavirus (HPV). As our knowledge about HPV infections has expanded, it has become possible to understand the course of RRP disease and unravel plausible efficient methods to manage the disease. However, the surge in reports on HPV has not been accompanied by a similar increase in research about RRP specifically. In this paper, we review the clinical manifestation and typical presentation of the illness. In addition, the pathogenesis and progression of the disease are described. On the other hand, we discuss the types of treatments currently available and future treatment strategies. The role of vaccination in both the prevention and treatment of RRP will also be reviewed. We believe this review is essential to update the general knowledge on RRP with the latest information available to date to enhance our understanding of RRP and its management.

## 1. Introduction

Recurrent Respiratory Papillomatosis (RRP) is a rare disease occurring in the upper aerodigestive tract [1]. It presents as a benign neoplasm that may arise in children and adults, giving a characteristic bimodal age distribution [2,3]. Based on age, the disease is classified into juvenile-onset RRP (JoRRP) and adult-onset RRP (AoRRP) [4]. In addition, a recent study suggested that the disease onset shows three peaks, specifically at ages 7, 35, and 64 [5]. Usually, the juvenile form of the disease is more aggressive and more likely to recur [3]. RRP disease incidence is higher in children and men. The number of cases of JoRRP compared to AoRRP varies based on the geographical area; while AoRRP is associated with Europe and South America, JoRRP is more common in Africa. Interestingly, the incidence of RRP is similar in both developed and developing countries, and disease severity is usually not affected by socioeconomic factors [4].

RRP is caused by human papillomaviruses (HPVs). HPV is a non-enveloped double-stranded circular DNA virus found in the *Papillomaviridae* family [6]. The HPV genome consists of the upstream regulatory region (URR), the non-coding region, in addition to the early and late harbor regions, which code proteins responsible for replication, transcription, and immune resistance [7]. The late (L) region is responsible for encoding the structural proteins L1-L2, and plays a role in virus assembly [8]. On the other hand, the early proteins E5, E6, and E7 act as oncogenes and are involved in altering molecular mechanisms of HPV-infected cells [9]. More than 200 distinct types of HPV are currently identified according to their genome and are classified as low and high-risk viruses. At least 17 high-risk types, including HPVs 16, 18, 31, 33, 35, 39, 45, 51, 52, 55, 56, 58, 59, 68, 73, 82, and 83, are reported to be involved in the onset and development of malignancies in cooperation with other oncogenes [10,11]. The E6/E7 proteins of high-risk HPVs have been found to enhance DNA integration into the host genome [12], plausibly due to enhanced chromosome rearrangements in high-risk HPV-infected cells, leading to mutation or/and deletion of both viral and host genes [13]. Moreover, E6/E7 proteins of high-risk HPVs can alter DNA repair pathways [12]. Indeed, high-risk HPVs, especially HPV16, have been found to play a pivotal role in vulvar cancer [14], uterine cervical cancer and its precancerous (CIN) lesions [15], in addition to anal [16] and oropharyngeal cancers [17]. On the other hand, low-risk HPVs (6 and 11) are rarely associated with cancer, and their infections result in the development of benign anogenital papillomas and skin warts [18,19]. Even though several distinct types of HPV may cause RRP, current evidence shows that the low-risk HPV types (HPV6 and 11) are the most common causal factors [20,21,22]. Nonetheless, high-risk HPV infection has also been reported to cause RRP [4]. A study by Stephen et al. [23] identified a similar role for E6/E7 of HPV11 (low-risk) in the stable maintenance of episomes as high-risk E6 and E7 proteins. In RRP-infected individuals, HPV DNA is usually episomal (HPV infects the basal cells and develops prolonged infection) instead of being fused or integrated with host DNA [4,24]. Studies have demonstrated that several microbial agents, including *G. vaginalis* and *Sneathia* spp. may facilitate HPV-induced infection, invasion, and multiplication [7,25]. In this regard, it is worth noting that there are three different licensed prophylactic HPV vaccines: the first generation includes two vaccines, quadrivalent (Gardasil) and bivalent (Cervarix); while the second generation is a nonavalent HPV vaccine (Gardasil 9). These vaccines were developed against HPV infections and HPV-induced diseases [26].

In adults, the main risk factor is increased sexual activity with multiple partners [3]. About 0.7% of mothers with HPV genital warts pass the disease to their infants. HPV DNA is detected in up to 80% of the neonates born to mothers with genital HPV [27]. The vertical transmission of HPV can be categorized into periconceptional (around the time of conception), prenatal (pregnancy), and perinatal (birth or immediately after that). It is known that juvenile-onset RRP is acquired during delivery by vertical transmission from the mother’s anogenital site to the child’s respiratory tract either through the birth canal or infected amniotic fluid and placenta [27]. A study by Venkatesan and colleagues demonstrated a significant risk (231 times more) of juvenile RRP in children born to mothers with active HPV infection than children born to unaffected mothers [28].

Moreover, previous investigations reported the possibility of transmission during the periconceptional period, as HPV is found in the male reproductive tract and semen samples, suggesting HPV transmission during fertilization [29,30]. Current evidence shows that the perinatal period is the most likely period for transmission [31]. Moreover, the risk of vertical transmission has also been linked with the mode of delivery. A meta-analysis concluded that cesarean section is associated with significantly lower rates of HPV transmission when compared to vaginal delivery [32]. Studies also reported childhood-onset RRP in firstborn and vaginally delivered children compared to patients of a similar age [33,34]. One of the plausible reasons includes a prolonged second stage of labor in primigravida women resulting in continual exposure of the fetus to the virus [35].

Nevertheless, a report by Zouridis et al. found no significant difference in vertical transmission when comparing vaginal delivery and cesarean section [36]. On the other hand, breastfeeding is also considered a mode of vertical transmission; a high prevalence of HPV subtypes, including HPV types 6 and 11, was found in the nipple and areola epithelia in breast cancer samples [37]. Meanwhile, an investigation revealed that most children have an absence of infection at six months, indicating temporary inoculation rather than actual vertical infection [38]. This indicates the need for further investigations to understand different aspects of RRP disease, including its etiological factors, development, and complication, leading to identifying the best treatment options.

## 2. Clinical Presentation

As mentioned above, most newborns are infected before or during delivery. Nonetheless, symptoms do not usually appear immediately; instead, they generally appear between 2 and 6 years. Patients infected with HPV type 11 have a younger presentation, aligning with the more aggressive course [4,39]. The most common early symptom is hoarseness, which might be challenging to notice at a young age [40]. Another initial symptom is progressive dysphonia, which is also more frequent in adults [22,41]. Due to upper airway involvement, dyspnea, chronic cough, recurrent upper respiratory infections, pneumonia, acute respiratory distress, dysphagia, and/or failure to thrive may be present [41,42]. In addition, presence of HPV genomic sequences has been reported in the nasal cavity [43]. These symptoms may lead to a misdiagnosis of asthma, laryngotracheobronchitis, foreign body aspiration, or laryngomalacia. Several reports show that the initial presentation relies heavily on the availability of healthcare services since countries and healthcare centers with poor facilities usually have a higher percentage of patients presenting with more severe symptoms like stridor and respiratory distress as the initial symptoms [44,45,46]. The initial site of infection is usually the larynx. Spread to extralaryngeal structures most commonly starts with the trachea, followed by the oropharynx, nasopharynx, nose, oral cavity, and rarely the lung, which can be identified using computed tomography (CT) [4]. This distribution is associated with poor innate immunity.

In addition to the delayed onset, there is also delayed diagnosis due to the subtle manifestations seen, causing diagnosis to occur about one year after initial symptoms appear [47]. Diagnosis is usually made using flexible fiberoptic laryngoscopy or direct laryngoscopy and biopsy in addition to the symptoms mentioned above [48]. Confirmatory diagnosis of RRP is done by laryngoscopy or fiberoptic bronchoscopy. Bronchoscopy is considered as the most reliable technique for diagnosis of RRP lesions of the central airway and aids in the therapeutic planning of RRP [3]. However, in cases of tracheobronchial changes, further evaluation is done using CT-scan [49]. While helical CT is the standard diagnosis method for RRP, it is infrequently diagnosed by X-ray [50]. In RRP patients including lung involvement, chest X-rays tend to indicate the presence of solid or cavitated pulmonary nodules [49]. On the other hand, helical CT demonstrates a higher accurate rate in the identification of tracheobronchial and pulmonary lesions [51]. Moreover, CT scan findings report the presence of focal or diffused airway narrowing on the mucosal surface [3]. CT scan images of lung involvement in RRP include single or multiple solid nodular or polypoid lesions mainly in the basal and posterior regions of varying sizes [3]. Other CT findings are associated with airway blocking, secondary infections as well as bronchiectasis [3]. A non-invasive technique for RRP diagnosis is virtual bronchoscopy of the tracheobronchial tree, providing three-dimensional images of the airway [52]. In addition, magnetic resonance imaging (MRI) shows presence of lesions in the larynx, tracheobronchial and pulmonary regions, however, its role in RRP diagnosis is not well-defined [53]. The presence and location of lesions, calcifications as well as association with pulmonary parenchyma abnormalities and clinical data indicate specific diagnosis and can help direct the therapeutic interventions [51,54]. Finally, histopathological findings confirm the diagnosis of RRP [54,55].

Histologically, RRP is composed of neoplastic squamous epithelium with exophytic, finger-like projections overlying supporting fibrovascular cores. The surface epithelium may show abnormal keratinization and basal cell hyperplasia. Epithelial atypia is usually absent [4,22,56]. Generally, papillomas appear as nodules or exophytic masses. Several papillamatous lesions grow at anatomical sites. Moreover, HPV is involved in epithelial maturation delay leading to thickening of the basal layer and increase in the number of nucleated cells in the suprabasal layer of the stratified epithelium [57]. In cases where lesions spread to the tracheobronical tree, the epithelium appears squamous, ciliated and cylindrical [49]. On the other hand, pulmonary lesions differ morphologically and appear as foci of squamous epithelium [49]. The lesions tend to grow, combine and damage the pulmonary parenchyma leading to cavity formation [49,51]. Histologically, although papillomas are benign, they can undergo malignant transformation and pathological changes include atypia focal necrosis, foci of keratinization and sheets of polyglonal tumor cells [53]. In addition, immunohistochemical analysis reports the presence of cytokeratins-5 and -6 demonstrating epithelial origin [53].

Staging is also necessary to determine the appropriate therapy. Staging includes an assessment of clinical features and a structural assessment. Remission is achieved in most patients with the support of a robust immune system, which plays an essential role in eliminating HPV-caused infections. Nevertheless, recurrence and persistence of the disease also may occur in several cases [4,58].

## 3. Immune Response

**Figure 1:**
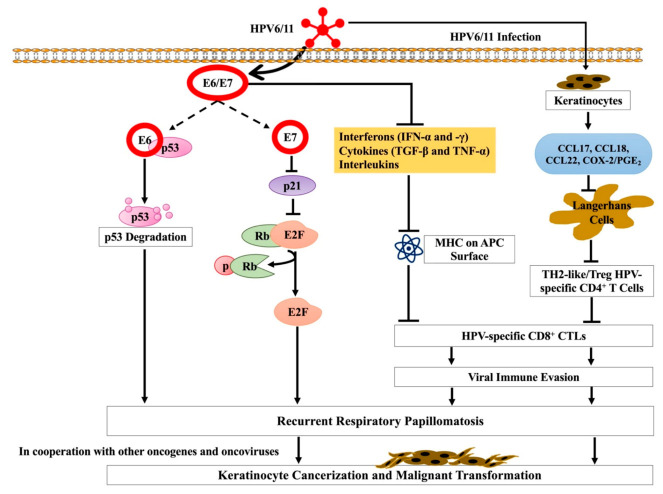
Outline of HPV type 6 and 11 infection pathways. Although RRP is not a malignancy, it can progress to cancer following further genomic deregulation caused by other oncogenes or oncoviruses.

It has been revealed that infants exposed to HPVs in the birth canal do not develop RRP, suggesting other factors, including immunologic and genetic factors affecting the development and recurrence of this disease (Figure 1) [28].

However, the pathogenesis of low-risk and high-risk HPVs is different [59]. While E6 and E7 proteins of low-risk HPV are involved in the evolved viral life cycle, they display a comparatively fewer transforming activities and do not play a role in genomic instability [59]. In contrast, E6 and E7 proteins of high-risk HPVs maintain a low copy number in infected cells with a prolonged persistence without causing clinical disease [59]. In addition, transformed cells influence the local immune environment allowing high-risk HPVs to escape from immune attack [59]. Several studies reported E6/E7 interactions with the host immune system; the E6/E7 protein of HPV inhibits activation of target human keratinocyte cells and transcription of inflammatory cytokines by deregulating the antiviral signaling cascade [60,61,62,63]. E6/E7 co-operate and inhibit the expression of several inflammatory cytokines, including interleukins (ILs)-2, -8, and -18 [60,64,65]. In RRP-infected patients, E6 can block the expression of interleukins (ILs), IL-2, and IL-8, thus, causing an imbalance in the adaptive immune response [66]. On the other hand, IL-18 plays a significant role in the skin inflammatory response to viral infection; increased IL-18 in the presence of HPV infection can reduce the cellular immune response [67]. A previous investigation reported that the CR3 region of E7 was required for triggering the production of IL-18 in keratinocytes; increased IL-18 expression inhibits the activation of CD4-positive lymphocytes [68]. The study indicates that elevated IL-18 can help HPV escape the immune response and contribute to HPV persistence in the epithelium [68]. Moreover, E6/E7 decrease antiviral interferon production by deregulating the roles of interferons (IFNs) regulatory factors (IRFs) [69,70] and nuclear factor kappa B (NF-κB) [71,72]. On the other hand, E7 inhibits cytostatic cascade of certain cytokines (transforming growth factor β (TGF-β), tumor necrosis factor α (TNF-α), IFN-α, IFN-γ, and insulin-like growth factors (IGFs)) involved in regulating cellular growth and immune reaction to viral infection. While E7 inhibits TGF-β, promoting epithelial cell growth [73], in keratinocytes expressing E7, TNF-β is deregulated, inducing cellular differentiation [74]. In addition, E7 reduces IFN-β expression and deregulates IFN-α–mediated cellular signaling, further evading immune response to IFNs [75,76]. In low-risk HPV-infected patients, response rate of IFNα is higher in comparison to those infected with high-risk HPVs [77], indicating involvement of high-risk HPVs in IFN signaling resistance. Additionally, the E6 protein of HPVs significantly impairs immune regulation in RRP [66], thus modifying immature Langerhans cells (iLCs) response and T_H_2-like/T_reg_ HPV-specific adaptive immunity [78]. A recent study in RRP patients showed compromised monocyte/ iLCs due to elevated levels of cyclooxygenase-2/prostaglandin E_2_ (PGE_2_) [78]. Another study in RRP patients reported a rise in a subset of circulating CD4^+^ T cells that expresses T_H_2-like cytokines, increasing the likelihood of a T_H_2 and T_reg_ response [79,80]. Additionally, in RRP patients, levels of several T_H_2-like chemokines (CCL17, CCL18, and CCL22) were elevated and associated with disease severity [81]. 

Additionally, the innate immune response is also dysregulated. This is partly due to the dysregulation of adaptive immunity, which leads to the dysfunctional activation of innate immunity. Increased IL-1F9 expression is found in patients with RRP and is associated with disease severity; IL-1F9 was found to induce a T_H_2-like response and modify innate signaling [82,83]. Interestingly, programmed death-ligand 1 (PD-L1), a negative immune regulator on APCs, is up-regulated in the papilloma and infiltrating immune cells [84]. These differences in immune responses may explain the reason behind the small percentage of people who develop clinical manifestations of the disease. The immune aspect of the disease has led to several trials aiming to use immune therapy to reduce the severity and/or occurrence of RRP [85,86].

## 4. Malignant Transformation

The risk of malignant transformation in RRP is exceptionally low (1–2%) [87]. In addition, squamous cell carcinomas that arise due to RRP are usually well-differentiated [88,89,90,91]. This risk is commonly found in adults with additional adverse factors [88,90,91]. Moreover, children with early-onset and prolonged disease carry a greater risk [28]. Interestingly, several studies have consistently found HPV-11 in malignant cases, suggesting a higher risk than HPV-6 [92,93,94,95]. Another study also identified HPV11-positive patients to develop malignancy in the area of the papilloma as compared to HPV6-positive patients, who showed no sign of malignant development [95]. In addition, a case series including HPV11-positive children with RRP reported the development of bronchogenic squamous cell carcinoma, indicating the possible transformation of RRP into malignancy [88,89,94,96].

As mentioned, the role of E6/E7 proteins of low-risk HPVs differs from high-risk HPVs as does their interaction with cellular proteins. While E6 proteins of both low- and high-risk HPVs bind to p53, only high-risk E6 proteins interact with the p53 core domain resulting in p53 degradation (Figure 1) [97]. Likewise, E7 proteins of both low and high-risk HPVs are also capable of cooperating with the Rb protein [98]. Nevertheless, E7 proteins of high-risk HPVs have a higher affinity for Rb which interrupts Rb and E2F interactions (Figure 1) [98]. A study by Zhang et al. [99] demonstrated that E7 proteins of both HPV6 (low-risk) and HPV16 (high-risk) can disrupt p130 as well as delay and/or reduce differentiation, however, as reported previously [98], only E7 protein of the high-risk HPV can control pRb and p107 for oncogenesis. In general, the E6/E7 proteins of high-risk HPVs play a causal role in oncogenesis. While, E6 is involved in telomerase activation and thus disrupts pathways involved in cell growth, proliferation, differentiation, immune recognition and survival signaling, E7 can promote genomic instability [100,101]. The E6/E7-induced activities of cell-cycle alteration, telomerase activation and genomic instability creates a suitable environment for cell transformation [101]. Several studies have also demonstrated a molecular mutation in the p53 oncogene to play a crucial role in malignant transformation [89,90,91,92]. E6 and E7 oncogenes of HPV target retinoblastoma (Rb) and p53, respectively, leading to keratinocyte cancerization and malignant transformation in cooperation with other oncogenes or oncoviruses (Figure 1) [93,102,103,104]. In some RRP cases, there are concurrent viral infections of RRP with other viruses, such as HSV-1, cytomegalovirus, and EBV [105,106]. In some instances, aggressive RRP can develop into lung cancer. A recent study conducted with Juvenile-onset recurrent respiratory papillomatosis (JoRPP) confirmed the involvement of E6 and E7 in RRP caused by low-risk HPV; higher levels of E6 and E7 transcription correlated with increased aggressiveness of the disease course [24]. Studies also revealed underlying molecular mechanisms of low-risk HPVs induced malignancies [82,103,107,108]. Expression of chemokine ligands CXCL1, CXCL6, CXCL8, and vascular endothelial growth factor (VEGF)-A were found to be differentially expressed in papillomas and to be associated with severe RRP and malignant progression [82,108]. In addition, the expression of both oncogenes, as well as tumor suppressor genes, also demonstrated differential expression in papillomas [82]. This suggests the use of E6 and E7 of HPV types 6 and 11 as possible prognostic biomarkers for RRP and a potential target for the treatment of this disease.

## 5. Treatment of RRP

Although there is no definitive cure for RRP, typical management includes surgical and medical therapy to treat RRP recurrence (Table 1). Table 1 summarizes the different therapeutic strategies used for RRP treatment.

### 5.1. Surgery

Surgery is the primary treatment of RRP; on an average basis, RRP patients undergo approximately four surgeries annually [141]. The purpose of surgery involves papilloma removal in parallel to preserving healthy respiratory tract tissue; common methods for surgery include microdebridement, sharp dissection, and Carbon dioxide (CO_2_)-laser [109,110]. Although microdebridement offers detailed papilloma resection, post-operative side effects can include vocal cord injury and scarring. CO_2_-laser surgery is the most frequently used laser for RRP [35]. CO_2_-laser surgery vaporizes the diseased tissue and helps minimize disease recurrence; however, cold steel excision micro instrumentation treatment is preferred over CO_2_-laser surgery to prevent scar formation of vocal cords, particularly in adults [35,85]. In addition, endoscopic microdebrider is preferred over CO_2_-lasers as it helps to rapidly reduce the papilloma in addition to improving voice quality, decreased mucosal injury, and better cost-benefit [111]. However, despite removing all clinically evident papilloma, latent viruses may remain in the nearby tissues.

Photodynamic Therapy (PDT) is another form of therapy used for RRP treatment that stimulates gradual necrosis of diseased tissue, causing the release of HPV oncoproteins (E6 and E7) [85]. This allows the presentation of antigens and short-term immunologic viral clearance interceded by IL-10 and IFN-γ [112]. Di-hematoporphyrin ether (DHE) was the first drug developed under PDT. The treatment showed small but statistically significant results in reducing RRP growth. However, patients treated with DHE became markedly photosensitive for 2-8 weeks following the treatment [35]. M-tetra(hydroxyphenyl) chlorine, the last drug developed, has shown comparatively fewer damaging results in animal models [35,112]. However, the dye utilized in PDT is not virus-specific and hence is likely to damage nearby healthy laryngeal tissue. 

On the other hand, the photoangiolytic laser provides a safe and effective treatment for RRP. The frequently used photoangiolytic laser is potassium titanium phosphate (KTP), a 585 nm flash dye or argon laser which disrupts papilloma microcirculation, thus shrinking the infected tissue. The KTP laser is often used alongside a ventilating bronchoscopy or ventilating resectoscope when the papilloma is present in the tracheobronchial tree [111]. In addition, since KTP laser provides surgical precision, it is used in combination with bevacizumab, an anti-angiogenic agent [113].

Although surgery is the primary treatment choice, in approximately 20% of the cases, adjuvant therapy is required [85,142]. The main indication for adjuvant therapy includes a total of more than four surgical procedures in 12 months or less, rapid regrowth of resected papilloma, or metastasis [35]. Some of the primary adjuvant therapies used are discussed in the section below.

### 5.2. Adjuvant Therapies

Adjuvant therapies include the use of interferon, antiviral therapeutics (Acyclovir, Cidofovir, Ribavirin), retinoids, in addition to inhibitors of the cyclooxygenase-2 [143]. 

Alpha-interferon was the first mode of treatment used in adjuvant therapy for RRP [114,115]. Interferons are proteins produced by cells to inhibit viral RNA and DNA replication by enhancing protein kinase and endonuclease [116]. However, the use of interferons induces both acute side effects, such as fever, nausea, fatigue, chills, or headache; Chronic side effects include a decrease in the child’s growth rate, increase in liver transaminase levels, and febrile seizures [35]. 

The use of other antivirals has also been encouraged for RRP treatment (Table 1). For example, Ribavirin is used for the treatment of a more aggressive laryngeal RRP [117]. Another antiviral drug, Acyclovir, was found effective in some RRP cases, with coexisting viral infections (HSV-1, cytomegalovirus, or EBV) [105,106]. The activity of this drug is dependent on the presence of virus-encoded thymidine kinase [118]. Cidofovir, an analog of cytosine, is either given intravenously, via nebulization, or by intralesional injection and is frequently used as an antiviral in adjuvant treatment for RRP [85,119]. Although intralesional administration has no local side effects, long-term risks have shown cidofovir to be carcinogenic [85].

Celebrex is a Cox-2-inhibitor that demonstrated anti papilloma activity, particularly in rabbit models [120]. Preliminary trials are ongoing to analyze the role of Celebrex in children and adults with RRP [121]. In addition, the measles-mumps-rubella (MMR) vaccine, when injected into RRP lesions, demonstrated moderate therapeutic benefit. However, further studies are needed to investigate the role of the MMR vaccine as a therapeutic agent for RRP (41,48). On the other hand, antireflux therapy is gaining importance as it has been implicated in reducing the recurrence rate of RRP [122,123,124]. Ranitidine (H2-antihistamine cimetidine) displayed immunomodulatory effects against HPV-induced RRP [123] and has been suggested as adjunctive therapy for RRP [124]. 

On the other hand, dietary supplements, including Indole-3-Carbinol (I3C) and retinoids, negative estrogen regulators, have been included as a treatment option for RRP, based on the elevated levels of estrogen binding present in RRP lesions [125]. A study in in-vivo models using immunocompromised mice treated with I3C showed a 75% reduction in the formation of HPV-induced papilloma lesions [126]. Hence, a plausible role of I3C in reducing RRP lesion size can be postulated. On the other hand, retinoids, metabolites, and vitamin A analogs were found to inhibit squamous differentiation (excess vitamin A) and hyperkeratinization (deficiency of vitamin A) [128]. In addition, Accutane (13-cis-retinoic acid) is being used for RRP treatment; however, it was found to have psychiatric and teratogenic side effects [127].

Treatment options for RRP also include gene therapies where target genes are present in diseased tissues and not normal cells (Table 1). In the context of RRP, targeting early HPV 6 and 11 genes, such as E2, E5, E6, and E7, might be particularly relevant. In addition, EGFR is expressed in RRP; hence use of EGFR inhibitors induced growth arrest and differentiation in HPV16-infected keratinocytes [129]. Moreover, gefitinib is proposed as a potential RRP treatment in the presence of extensive tracheobronchial epithelial cells [130]. Additionally, the use of topical aerosol EGFR inhibitors has been reported to enhance epithelial differentiation and inhibit RRP growth [35].

In addition to gene therapy, checkpoint and VEGF inhibitors are also being studied for RRP treatment (Table 1). Avastin (Bevacizumab), a monoclonal antibody against vascular endothelial growth factor (VEGF), has been found to increase the time between surgical interventions and reduce RRP disease severity [133]. Moreover, intravenous administration of Avastin is used as adjuvant therapy in patients with advanced pulmonary and tracheal RRP [131,132,134]. Furthermore, another monoclonal antibody, Avelumab (Bavencio), an anti-PD-L1 monoclonal antibody, was tested in RRP patients; all patients with laryngeal RRP responded to the treatment [144], indicating the use of PD-L1 inhibitors as an additional alternative adjuvant therapy.

### 5.3. HPV Vaccines

In addition to the above therapeutic strategies, HPV vaccinations are involved in RRP preventive treatment (Table 1 and Table 2). The vaccines are prepared using recombinant technology established on the self-assembly characteristic of the L1 capsid protein of the virus [26]. Although the vaccines consist of viral-like particles, they are not infectious. Most importantly, HPV vaccines differ in the number of HPV types they are comprised of and their targets [26]. Table 2 summarizes the comparison between the three HPV vaccines.

A clinical trial conducted in 2005 included 27 children with severe RRP, used HspE7 (recombination fusion product) as a treatment agent. It reported a significant (93%) increase in the first inter-surgical interval [146].

On the other hand, the quadrivalent HPV vaccine, Gardasil-4 (Merck, Whitehouse Station, NJ, USA) that targets HPVs 6, 11, 16, and 18 and the nonavalent HPV vaccine, Gardasil -9 that targets HPVs 6, 11, 16, 18, 31, 33, 45, 52 and 58 have been postulated for RRP treatment [136,137,138]. Recently, Rosenberg et al. performed a systematic review and meta-analysis of 12 publications and 63 RRP patients treated with Gardasil-9 [138]. The meta-analysis reported a significant decrease in surgeries per month after the vaccination [138]. Although the underlying mechanism for the therapeutic effect of Gardasil-9 is not known, it is designed to target the L1 protein of the HPV capsid to generate neutralizing antibodies and create a vaccine-induced humoral response, thus, inhibiting HPV infection within or around the surgical site [135,140]. In addition to Gardasil-9, novel DNA vaccines aim to target the E6/7 oncoproteins of HPV and create a significant T-cell immunologic response [140]. The novel INO-3016 vaccine demonstrated immunologic response both in-vitro and in-vivo and reduced inter-surgical interval [139,140]. 

Furthermore, an HPV-DNA test can act as a predictor of RRP lesion recurrence. If a patient tests negative, he is less likely to experience recurrence than other patients who consistently test positive [138]. Since RRP patients display a significant immunologic response to HPV vaccination, HPV vaccines can be considered a therapeutic option for RRP-infected individuals.

## 6. Conclusions

In conclusion, RRP is generally a benign disease characterized by papillomatous lesions in the aerodigestive tract, commonly affecting infants and young adults. However, RRP can recur and spread within the respiratory tract, leading to mortality. The underlying etiology, clinical presentations, and diagnosis are essential for choosing treatment options. The review indicates that in addition to surgery, RRP incidence is reducing with the increased use of HPV vaccines, especially Gardasil 4 and 9. Ongoing research using adjuvant therapy and DNA vaccines, along with PD-L1 inhibitors may pave the way for additional therapeutic strategies.

## Figures and Tables

**Table 1:** Therapeutic strategies used in RRP treatment.

Therapy	Treatment	Rationale	Reference
Surgery	Microdebridement	Removal of papilloma along with maintaining healthy respiratory tract tissue	[109,110]
	Sharp dissection		
	CO_2_ laser surgery		
Photodynamic Therapy	Di-hematoporphyrin ether (DHE)	Short-term immunologic viral clearance of antigens mediated by IL-10 and IFN-γ on presence of E6/E7 oncoproteins of HPV due to necrosis of infected tissue	[85,111,112]
	M-tetra(hydroxyphenyl) chlorine		
Photoangiolytic Laser	Potassium titanium phosphate [KTP]	Reduces the infected tissue by disrupting papilloma microcirculation	[111,113]
Adjuvant Therapies	Alpha-interferon	They block viral RNA or DNA replication by stimulating protein kinase and endonuclease	[114,115,116]
	Antiviral Therapies		
	Ribavirin	Used to treat respiratory syncytial virus in infants. Used for treatment of aggressive laryngeal RRP	[117]
	Acyclovir	Treatment of RRP with co-existing viruses (HSV-1, EBV or cytomegalovirus) as it targets thymidine kinase presented by these viruses	[105,118]
	Cidofovir	An analog of cytosine, it reduces DNA transcription efficacy	[119]
	Celebrex (Ongoing Clinical Trials)	It is a COX-2 inhibitor and aims to provide a prolonged inhibitory effect on microvascular regrowth and COX-2 enzyme, thus preventing recurrence of RRP	[120,121]
	Ranitidine	Demonstrates immunomodulatory effects and reduces RRP recurrence	[122,123,124]
	Dietary Supplements		
	Indole-3-Carbinol (I3C)	Increases estrogen binding in RRP lesions. In mice, I3C reduced formation of papilloma lesion by 75%	[125,126]
	Retinoids, metabolites and Vitamin A	Excess and lack of vitamin A reduces squamous differentiation and induces hyperkeratinization	[127,128]
	Inhibitors		
	EGFR inhibitors (Gefitinib)	They inhibit epithelial growth and differentiation of HPV16-infected keratinocytes, thus reducing RRP growth. Used in RRP treatment in the presence of extensive tracheobronchial epithelial cells	[129,130]
	VEGF inhibitors	Inhibits VEGF activity and prevents receptor activation, thus increasing time between surgical intervention and reduces RRP severity	[131,132,133,134]
Vaccines	HPV Vaccines		
	Cervarix	Targets HPVs-16 and -18	[135,136,137,138]
	Gardasil	Targets HPVs-6, -11, -16 and -18	
	Gardasil-9	Targets HPVs-6, -11, -16, -18, -31, -33, -45, -52 and -58	
	DNA Vaccines		
	INO-3016	Targets the E6/E7 oncoproteins of HPV, creates a T-cell immunological response and reduces surgical intervention	[139,140]

CO_2_: Carbon dioxide; COX-2: Cyclooxygenase-2; DHE: Di-hematoporphyrin ether; EBV: Epstein–Barr virus; EGFR: Epidermal growth factor; HPV: Human papillomavirus; HSV-1: Human simplex virus-1; I3C: Indole-3-Carbinol; IFN-γ: Interferon gamma; IL-10: Interleukin-10; KTP: Potassium titanium phosphate; RRP: Recurrent respiratory papillomatosis; VEGF: Vascular endothelial growth factor.

**Table 2:** Comparison between the three prophylactic HPV vaccines [26,145].

	Bivalent Vaccine	Quadrivalent Vaccine	Nonavalent Vaccine
Brand Name	Cervarix	Gardasil	Gardasil-9
Type of Viral-Particle	HPV-16, -18	HPV-6, -11, -16, -18	HPV-6, -11, -16, -18, -31, -33, -45, -52 and -58
Expression System	Baculovirus-insect cell	Yeast	Yeast
Adjuvant System	AS04 adjuvant system in sodium chloride, sodium dihydrogen phosphate dihydrate	Amorphous Aluminum Hydroxyphosphate Sulfate	Amorphous Alumi-num Hydroxyphosphate Sulfate
Recommended Dose	20/20 μg	20/40/40/20 μg	30/40/60/40/20/20/20/20/20 μg
Scheduled Dose	0, 1 and 6 months	0, 2 and 6 months	0, 2 and 6 months

## Data Availability

Not applicable.

## Abbreviations

AoRRP	Adult-onset RRP
APC	Antigen-presenting cell
CCL	Chemokines
CO2	Carbon dioxide
COX-2	Cyclooxygenase-2
CT	Computed tomography
CXCL	Chemokine ligand
DHE	Di-hematoporphyrin ether
EBV	Epstein–Barr virus
EGFR	Epidermal growth factor receptor
HPV	Human papillomavirus
HSV-1	Herpes simplex virus-1
I3C	Indole-3-Carbinol
IGF	Insulin-like growth factors
IHC	Immunohistochemistry
IL	Interleukin
iLCs	Immature Langerhans cells
INF	Interferon
IRF	Interferons regulatory factors
JoRRP	juvenile-onset
MMR	Measles-mumps-rubella
KTP	Potassium titanium phosphate
MRI	magnetic resonance imaging
NF-κB	Nuclear factor kappa B
PGE2	Prostaglandin E2
PD-L1	Programmed death-ligand 1
RRP	Recurrent respiratory papillomatosis
TGF	Transforming growth factor
TH2	T helper type 2
TNF	Tumor necrosis factor
Treg	Regulatory T cells
VEGF	Vascular endothelial growth factor
